# An empirical evaluation of the SF-12, SF-6D, EQ-5D and Michigan Hand Outcome Questionnaire in patients with rheumatoid arthritis of the hand

**DOI:** 10.1186/s12955-016-0584-6

**Published:** 2017-01-24

**Authors:** Melina Dritsaki, Stavros Petrou, Mark Williams, Sarah E. Lamb

**Affiliations:** 1 Nuffield Department of Orthopaedic Rheumatology and Musculoskeletal Sciences, University of Oxford, Oxford, UK; 2 Division of Health Sciences, Warwick Medical School, University of Warwick, Coventry, UK; 3 Department of Sport and Health Sciences, Oxford Brookes University, Oxford, UK

**Keywords:** Utility measures, Outcome assessment, Measurement properties, Health Economics, Rheumatoid arthritis, EQ-5D, SF12, SF-6D

## Abstract

**Background:**

The aim of this study was to assess the psychometric properties, namely acceptability, validity, reliability, interpretability and responsiveness of the EuroQol EQ-5D (EQ-5D visual analogue (VAS) and EQ-5D (utility)), Short Form 12 Dimensions (SF-12), SF-6D and Michigan Hand Outcome Questionnaire (MHQ) in patients with rheumatoid arthritis (RA) of the hand.

**Methods:**

The empirical investigation was based upon data from a randomised controlled trial of 488 adults with rheumatoid arthritis who had pain and dysfunction of the hands and/or wrists. Participants completed the EQ-5D, SF-12 and MHQ at baseline and at 4 and 12 months follow up. Acceptability was measured using completion rates over time; construct validity using the “known groups” approach, based on pain troublesomeness; convergent validity using spearman’s rho correlation (ρ); reliability using internal consistency (Cronbach’s alpha); interpretability using minimal important differences (MID); and responsiveness using effect sizes and standardised response means (SRM) stratified by level of self-rated improvement in hand and wrist function or level of self-rated benefit and satisfaction from trial treatments.

**Results:**

At baseline, the study population had a mean age of 62.4 years, a mean MHQ score of 52.1 and included 76% women. The EQ-5D (utility) had the highest completion rates across time points. All instruments discriminated between pre-specified groups based on pain troublesomeness. Convergent validity analysis indicated that the MHQ score correlated strongly with the EQ-5D (ρ = 0.65) and SF-6D (ρ = 0.63) utility scores. The MHQ was most responsive at detecting change in indicators of RA pain severity between baseline and 4 months, whilst minimal important differences varied considerably across PROMs.

**Conclusions:**

The instruments evaluated in this study displayed varying psychometric properties in the context of RA of the hand. The selection of a preferred instrument in evaluative studies should ultimately depend on the relative importance placed on individual psychometric properties and the importance placed on generation of health utilities for economic evaluation purposes.

## Background

Rheumatoid arthritis (RA) is a chronic and unpredictable disorder that can cause persistent joint pain, joint damage and long-term disability (especially in the hands and feet). The economic cost of RA is substantial for individual patients, health services and society as a whole [1]. Patients with poor and declining function from their diagnosis of RA generate elevated medical care costs [2]. A report by the National Rheumatoid Arthritis Society (NRAS) in 2010 found that the overall cost of RA to the UK economy was almost £8 billion per annum with National Health Service (NHS) expenditure totalling approximately £700 million per annum [3].

Patient reported outcome measures (PROMs) are increasingly used to measure health-related quality of life (HRQoL) from the patient perspective. PROMs have also increasingly been used in randomised controlled trials (RCTs) and other evaluative studies to measure the benefits of interventions in terms of health status or HRQoL. PROMs can be condition-specific or generic. Generic PROMs can either be preference-based (patient responses are generally used to generate profile scores, which are converted into index scores based on preferences for a given health state) or non-preference based (patient responses are generally summed to provide a score) with examples of the former such as the EQ-5D and SF-6D offering several advantages [4, 5]. The main advantages of generic preference-based PROMs are their ease of administration and high rate of completion, the generalizability of their results and their ability to meet the requirements of decision-making bodies, such as the National Institute for Health and Care Excellence (NICE) in England and Wales, concerned with cost-effectiveness comparisons [6]. Furthermore, preference-based outcome measures can incorporate the impact of treatment or ill health within multidimensional scales and can be combined with data on survival in the form of quality-adjusted life years (QALYs) [4]. Given the increasing diffusion of PROMS within evaluative research and the increasing use of the outputs of preference-based outcome measures within decision-making processes, it is important to establish the relative merits of alternative PROMS in specific clinical and research contexts.

To be useful in assessing HRQoL in individuals with RA of the hand, PROMS should satisfy a range of psychometric properties. The psychometric literature has developed a number of criteria to judge the performance of different instruments, key to which are acceptability, validity, reliability, interpretability and responsiveness to changes in health state. Although the construct validity of the EQ-5D has been investigated in the context of RA [7] and the responsiveness of the EQ-5D and SF-6D in patients with early arthritis [8], only one previous study has compared the psychometric properties of generic HRQoL measures (HUI2, HUI3, SF-6D and EQ-5D) with a disease-specific instrument (Rheumatoid Arthritis Quality of Life Scale, RAQoL) in the RA context [9]. The Michigan Hand Outcome Questionnaire (MHQ) is a well-established measure for patients with RA and widely used in clinical trials [10]. It has previously been compared to the Health Assessment Questionnaire (HAQ) in patients with RA [11] and also compared to the SF-12 in a study including patients with thumb osteoarthritis [12]. However, no studies have, to our knowledge, have so far investigated the performance of the MHQ in relation to preference-based measures..

The current study aims to fill this research gap by investigating the psychometric properties and performance of generic PROMs compared to the MHQ for patients with RA of the hand. It is anticipated that the results will provide evidence for the use of generic (EQ-5D, SF-12, SF-6D) and condition-specific PROMs in future research studies, including economic evaluations, related to RA.

## Methods

### SARAH trial

The SARAH trial was a pragmatic, multi-centre, randomised controlled trial conducted with 1 year follow-up. 488 participants with RA who had pain and dysfunction of the hands and/or wrists were randomised to either a tailored exercise programme in addition to usual care (*n* = 246) or to usual care alone (*n* = 242).

The primary method of data capture was face-to-face research clinic appointment.. Baseline and follow-up data at 4 and 12 months after randomisation was collected, including the MHQ score, EQ-5D utility, EQ-5D VAS, SF-12 and SF-5D at each of these time points.. Further details about the SARAH trial, its sampling procedures, methodology, outcome measures and responses rates are reported in full elsewhere [13]. Since we were primarily interested in the properties of the outcome measures used, rather than any evaluation of the interventions in the trial, all SARAH participants were included in the analyses reported here, regardless of trial allocation. The SARAH trial was approved by the Oxford C Multicentre Research Ethics Committee in June 2008.

### Patient reported outcome measures

#### MHQ

The primary outcome measure for the SARAH study was the MHQ overall hand function score at 12 months. The MHQ is a common hand-specific outcome measurement tool for patients with chronic hand conditions [14]. The MHQ has been validated for use in a wide range of patient samples. More specifically, it has been used in carpal tunnel syndrome [15, 16], distal radius fracture [17], reconstruction [18, 19] and arthroplasty in RA [20, 21]. The MHQ is appropriate for use in RA populations due to the comprehensive information gathered on functional abilities as well as patient satisfaction, pain and hand appearance. It has been utilised to assess disability and it is often an outcome measure for clinical trials in RA. It measures patient perception of hand function, appearance, pain, and satisfaction. It is intended for people with hand or wrist conditions or injuries [14]. It can be used to measure a patient’s general hand function, or can be used to assess changes in hand function over time, e.g. pre- and post-operation. It consists of 37 items and 6 subscales: overall hand function, activities of daily living (ADL), pain, work performance, aesthetics, and patient satisfaction with hand function. Scores range from 0 to 100, with higher scores indicating better performance, except for the pain scale. For the pain scale, a higher score indicates more pain [14].

#### EQ-5D

The EQ-5D-3L [22] (hereafter EQ-5D for brevity) comprises two components that assess health status on the day of completion. The first component is a self-reported descriptive system with five health dimensions (mobility, self-care, pain/discomfort, usual activities, and anxiety/depression) each divided into three different levels, namely no problems, some or moderate problems and severe or extreme problems. Responses to the descriptive system are generally valued using the time-trade method. For the purposes of this study, we applied the York A1 (Dolan) tariff set derived from a survey of the UK general population (*n* = 3337), which used the time trade-off valuation method to estimate utility scores for a subset of 45 EQ-5D health states, with the remainder of the EQ-5D health states subsequently valued through the estimation of a multivariate model [23]. Resulting utility scores range from -0.59 to 1.0, with 0 representing death and 1.0 representing full health, with some health states considered worse than death (<0). A further component of the EQ-5D consists of a visual analogue scale (VAS), which asks people to rate their current overall health on a scale from 0 (the worst health state they can imagine) to 100 (the best health state they can imagine).

#### SF-12 and SF-6D

The SF-12 consists of 12 items that assess 8 dimensions of health: physical functioning, role-physical, bodily pain, general health, vitality, social functioning, role-emotional and mental health. The SF-12 was scored as described by Ware [24]. The SF-12 measures various aspects of physical and mental health from which physical and mental summary scores can be calculated. The Physical Component Summary Score (PCS) and Mental Health Component Score (MCS) are both standardised to have a mean of 50 and a standard deviation of 10 [13].

A derivative of the SF-12 is the SF-6D, which is a multi-attribute utility measure composed of 6 dimensions (physical functioning, role limitation, social functioning, pain, energy, mental health), each of which has between four to six levels. The SF-6D generates 18,000 possible health states. To estimate health utilities for the SF-6D, we applied an algorithm developed by Brazier and colleagues [5] who surveyed a representative sample of the UK general population using the standard gamble technique. Utility values for SF-6D health states can fall between 0.30 and 1.0, where 1.0 represents full health and 0 represents death.

### Indicators of RA pain

In the SARAH trial, RA pain was measured using the Troublesomeness questionnaire (range 0-20, higher score indicates greater pain) [25] at baseline, and at 4 and 12 months post-randomisation.

### Statistical analysis

We followed the definitions and recommendations from the COSMIN (Consensus-based Standards for the selection of health Measurement Instruments) checklist [26], alongside a previously published checklist of assessment criteria for PROMs [27], when analysing the psychometric properties of the MHQ, EQ-5D (utility) (preference-based responses to the EQ-5D descriptive system), EQ-5D (VAS), SF-12 and SF-6D in the SARAH trial. Statistical analysis was conducted using STATA version 13.0 (Stata Corporation, Texas, USA) [28].

#### Acceptability

A PROM must be practical and acceptable to the population that will be completing the instrument and also represent the interests and perspectives of many different individuals associated with the PROM. The acceptability of the different study PROMs was measured using completion rates at baseline and each of the two follow up time points (4 and 12 months post randomisation) [29, 30].

#### Validity

The validation process for PROMs aims to establish whether a measure is useful in reaching the objective it has been developed for. The overall validity of an instrument is composed of a number of important components, such as content, construct and criterion validity. From a theoretical point of view a perfect validation process would compare the outcomes of the examined instrument to an external “gold standard”. However, for a number of abstract constructs such as pain, happiness or HRQoL, an external gold standard does not exist. This has led to the development of indirect empirical tests of validity [31]. Although many different indirect empirical tests of validity have been proposed in the social science literature, we focussed on construct and convergent validity..

Construct validity concerns the degree to which the scores of an instrument are an adequate reflection of the dimensionality of the construct to be measured [26]. The construct validity of the instruments used in the SARAH trial was assessed by the “known groups” approach [32]. In known groups validity, we take pre-specified groups where we would expect there to be a difference in health status, and thus instrument scores. The different scores between groups for alternative measures can then be compared to see if there is a pattern in the sensitivity to these expected differences [33]. Independent samples t-tests were performed to estimate the ability of each summary score, i.e. MHQ), EQ-5D (utility), EQ-5D (VAS), SF-12 (PCS), SF-12 (MCS) and SF-6D utility, to discriminate between groups with different RA severity at baseline. RA pain severity was measured using the Troublesomeness questionnaire () [25].We classified individuals according to their pain troublesomeness using a 30% threshold (low: pain troublesomeness score <30%; high: pain troublesomeness ≥30%) [25]. To assess convergent (discriminant) validity we assessed the relationship between continuous clinical (MHQ)) and health-related utility measures (EQ-5D utility, SF-6D utility) at baseline with the Spearman’s rank correlation coefficient. A correlation coefficient between 0.9 and 1.0 suggests that variables can be considered very highly correlated. Correlation coefficients between 0.7 and 0.9 indicate variables that can be considered highly correlated. Correlation coefficients between 0.5 and 0.7 suggests that variables can be considered moderately correlated, whilst correlation coefficients between 0.3 and 0.5 indicate variables that have a low correlation [34].

#### Reliability

In the current study, we evaluated one type of reliability, internal consistency for the MHQ EQ-5D(utility), EQ-5D (VAS), SF-12 (PCS), SF-12 (MCS) and SF-6D at baseline. Internal consistency reliability measures the homogeneity of the items comprising a scale; that is, whether the items in the same scale measure the same underlying concept. We used the Cronbach’s alpha (α) coefficient to express internal consistency. Cronbach’s alphas can range from 0 to 1.0, where 1.0 indicates perfect internal consistency. Generally, consistency is considered unacceptable for α <0.5, poor for 0.6 > α ≥ 0.5, questionable for 0.7 > α ≥ 0.6, acceptable for 0.8 > α ≥ 0.7 and good for 0.9 > α ≥ 0.8 [32]. Values > 0.90 indicate redundancy [34].

#### Interpretability

Interpretability is defined as the degree to which one can assign qualitative meaning to an instrument’s quantitative scores or change in scores [26]. Although not generally considered as a psychometric property, it is an important characteristic of a measurement instrument. The interpetability of the different study PROMs was measured using the minimal important difference (MID), which reflects the smallest amount of change in a score that is meaningful to a patient [36].

#### Responsiveness

Responsiveness considers whether the changes registered by a measure over time correspond to those expected based on an external reference measure of health [35]. We made use of two different reference measures to estimate the responsiveness of all study PROMs. The first referent was participant self-rated improvement in their hands and wrists, which used a seven- point Likert scale asking whether they had completely recovered, were much improved, slightly improved, showed no change, were slightly worse, much worse or vastly worse. These were collapsed into three categories, namely improved, no change and worsened for the purposes of these analyses. The second referent was a self-rated measure of benefit and satisfaction from trial treatments that assessed whether participants experienced substantial benefit, moderate benefit, no benefit, moderate harm or substantial harm. These were collapsed into three categories, namely benefit, no benefit and harm for the purpose of these analyses. The estimate of responsiveness was measured from baseline to 4 months and 4 months to 12 months for the self-reported hand and wrist functioning measure, and from baseline to 4 months and baseline to 12 months for the measure of benefit and satisfaction from trial treatments. A number of statistical tests were employed for this purpose, including the Effect Size (ES) and Standardize Response Mean (SRM) The ES can be defined as the change in mean score divided by the standard deviation of the instrument scores at baseline. The SRM divides the mean change in score by the standard deviation of individuals’ change in score. Changes in both were considered large when the ES and SRM were greater than 0.8, moderate when they were between 0.79 and 0.5 and small when they were between 0.49 and 0.2 [26].

## Results

A total of 488 participants were recruited into the SARAH trial, 452 (92%) and 438 (89%) of whom were followed up at 4 and 12 months, respectively. At inclusion in the study there were 76% females and the mean age was 62.4 years (Table 1).

**Table 1:** Baseline characteristics and response rates to each outcome measure at each time point (*n* = 488)

	Baseline (*n* = 488) (mean, SD)	4 months (*n* = 452)	12 months (*n* = 438)	Complete responders (all time points)
Age: mean (SD)	62.4			
Sex: % female	76%			
Disease duration (years)	10			
MHQ	100% (52)	92%	90%	87%
EQ-5D (utility)	99.21% (0.58,0.27)	100%	98.93%	99.10%
EQ-5D (VAS)	100% (66.71,18.75)	92.42%	88.93%	86.27%
SF-12 (PCS)	97% (34,9.67)	87%	84%	76%
SF-12 (MCS)	97% (49,10.99)	87%	84%	76%
SF-6D	99% (0.63,0.14)	89%	86%	81%

### Acceptability

Response rates for each outcome measure at baseline, and 4 and 12 months follow up are reported in Table 1. Response rates across all study time points ranged from 76.0% (SF-12) to 99.1% (EQ-5D utility). The SF-6D response rate (80.1%) was slightly higher than for both SF-12 subscales (76.0%). At baseline there were no missing data for the MHQ and the EQ-5D (VAS).

### Validity

Table 2 shows the results of the known–groups validity tests at baseline. Although all differences between low and high pain troublesomeness scale scores were statistically significant at the 5% significance level, not all instruments discriminated well between patients who had RA pain versus those who did not (as depicted by the pain troublesomeness scale). The MHQ and SF-12 (PCS) had large effect sizes (>0.8), while the remainder of the instruments had medium effect sizes.

**Table 2:** Known- groups (construct) validity effect sizes for the pain troublesomeness (baseline data)

Instruments	Pain Troublesomeness		
	Low (*n* = 126)	High (*n* = 362)	Effect size (95% CI)
	Mean HRQoL score (SD)		
MHQ	66.23(13.76)^a^	45.34 (13.95)	**1.51** (1.28,1.73)
EQ-5D (utility)	0.73 (0.20)^a^	0.53 (0.28)	0.77 (0.57, 0.98)
EQ-5D (VAS)	77.10(15.67)^a^	63.09(18.41)	0.79 (0.58,0.99)
SF-12 (PCS)	40.70(9.67)^a^	31.81(8.57)	**0.81** (0.60, 1.02
SF-12 (MCS)	51.99(9.10)^a^	47.33(11.35)	0.33 (0.13, 0.54)
SF-6D	0.71(0.13)^a^	0.61(0.13)	0.78 (0.57, 0.99)

Statistically significant differences between^a^ Low/ High score of pain troublesomeness (2 sample *t*-test). Effect size is calculated as the difference in means scores divided by the pool standard deviations. Effect size values for the dichotomous variables are considered small (<0.5), medium (0.5-0.8), or large (>0.8). Values in bold indicate large effect sizes

Table 3 presents the spearman’s rho correlation coefficients between the various instruments with all them being statistically significant at the 1% level of significance. Our results suggest that the MHQ correlated moderately with the SF-6D (ρ = 0.63) and EQ-5D (ρ = 0.65).

**Table 3:** Convergent (discriminant)validity. Mutitrait-multimethod (MTMM) correlation matrix illustrating the correlation of the different measures at baseline, missing data excluded pairwise for each comparison (*n* = 488)

	MHQ (overall)	MHQ (hand function both)	EQ-5D (utility)	EQ-5D (VAS)	SF-12 (PCS)	SF-12 (MCS)	SF-6D
MHQ	1						
EQ-5D (utility)	0.65	0.48	1				
EQ-5D (VAS)	0. 51	0. 42	0. 57	1			
SF-12 (PCS)	0. 59	0. 46	0. 61	0. 56	1		
SF-12 (MCS)	0. 32	0. 23	0. 42	0. 40	0. 25	1	
SF-6D	0.63	0.42	0.68	0.58	0.70	0.67	1

Correlation coefficients (spearman’s rho). All correlations are statistical significance (*p* < 0.01). Values in bold indicate correlations expected to exceed 0.7 (convergent validity)

### Reliability

The internal consistency of the study outcome measures as estimated by the Cronbach’s alpha coefficient at baseline (Table 4) was similar across all scales and above the threshold of 0.70 recommended for broader use in clinical research [10].

**Table 4:** Average inter-item correlation and Cronbach’s Alpha scores for study outcome measures at baseline

	Average Inter- item correlation	Cronbach’s Alphas	Internal Consistency
MHQ	0.45	0.83	Good
EQ-5D (utility)	0.47	0.84	Good
EQ-5D (VAS)	0.48	0.84	Good
SF-12 (PCS)	0.46	0.84	Good
SF-12 (MCS)	0.52	0.87	Good
SF-6D	0.44	0.83	Good

Internal consistency is considered unacceptable for α <0.5, poor for 0.6 > α ≥ 0.5, questionable for 0.7 > α ≥ 0.6, acceptable for 0.8 > α ≥ 0.7, good for 0.9 > α ≥ 0.8 and excellent for α ≥ 0.9 [32, 34]

### Responsiveness and Interpretability

Mean scores for each PROM at baseline and 4 months follow-up (Table 5) and at 4 and 12 month follow-up (Appendix 1) are shown for the self-reported hand and wrist functioning measure, which was used to estimate responsiveness; changes over time and ES and SRM estimates are also presented.

**Table 5:** Responsiveness of measures over time to self-reported hand and wrist functioning; baseline to 4 months

	n	T_0_	T_4_	Δ (95% CI)	SRM (95% CI)	ES (95% CI)
MHQ						
Improved	177	51.66	64.79	13.13 (9.67 to 16.58)	0.56 (-1.88 to 3.00)	0.79 (-1.64 to 3.23)
No change	150	54.15	56.97	2.82 (-0.96 to 6.59)	0.12 (-2.55 to 2.79)	0.16 (-2.51 to 2.83)
Worsened	113	47.2	45.37	-1.83 (-6.03 to 2.36)	-0.08 (-3.05 to 2.89)	-0.11 (-3.08 to 2.85)
EQ-5D (utility)						
Improved	177	0.58	0.69	0.11 (0.06 to 0.16)	0.31 (0.27 to 0.36)	0.44 (0.41 to 0.49)
No change	150	0.61	0.62	0.01 (-0.04 to 0.06)	0.03 (-0.01 to 0.07)	0.04 (0.00 to 0.08)
Worsened	113	0.55	0.48	-0.07 (-0.15 to 0.01)	-0.16 (-0.22 to -0.11)	-0.23 (-0.28 to -0.18)
EQ-5D (VAS)						
Improved	177	68.28	75.78	7.5 (3.9 to 11.10)	0.31 (-2.38 to 3.00)	0.43 (-2.26 to 3.13)
No change	150	68.99	67.46	-1.53 (-5.67 to 2.62)	-0.05 (-2.87 to 2.76)	-0.08 (-2.90 to 2.73)
Worsened	113	64.92	60.43	-4.49 (-9.50 to 0.52)	-0.16 (-3.81 to 3.48)	-0.23 (-3.88 to 3.41)
SF-12 (PCS)						
Improved	177	34.59	36.63	2.04 (-0.5 to 4.58)	0.12 (-1.44 to 1.67)	0.19 (-1.71 to 2.11)
No change	150	33.13	34.48	1.35 (-1.31 to 4.01)	0.081 (-1.89 to 2.05)	0.11 (-1.85 to 2.08)
Worsened	113	31.43	30.44	-0.99 (-3.83 to 1.86)	-0.06 (-2.04 to 1.91)	-0.09 (-2.06 to 1.88)
SF-12 (MCS)						
Improved	177	48.98	48.12	-0.86 (-3.69 to 1.98)	-0.04 (-1.75 to 1.66)	-0.06 (-1.77 to 1.65)
No change	150	46.52	49.29	2.77 (-0.44 to 5.98)	0.13 (-2.34 to 2.62)	0.19 (-2.29 to 2.68)
Worsened	113	46.02	44.01	-2.01 (-5.76 to 1.74)	-0.09 (-2.75 to 2.55)	-0.14 (-2.79 to 2.51)
SF-6D						
Improved	177	0.65	0.68	0.03 (-0.00 to 0.06)	0.15 (0.12 to 0.16)	0.21 (0.19 to 0.23)
No change	150	0.65	0.65	0 (-0.03 to 0.03)	0 (-0.02 to 0.02)	0 (-0.02 to 0.02)
Worsened	113	0.6	0.59	-0.01 (-0.05 to 0.03)	-0.05 (-0.07 to -0.02)	-0.07 (-0.09 to -0.04)

There was a statistically significant change in MHQ score for patients reporting improved hand and wrist functioning (Δ = 13.13) between baseline and 4 months. This was also the case for the EQ-5D (utility) (Δ = 0.11) and EQ-5D (VAS) (Δ = 7.5) (Table 5). Minimally important differences (MID) [interpretability] for each PROM varied; whilst a meaningful alteration in MHQ score required a large change over the study period, all other measures required smaller numerical changes. Table 5 summarises the ESs and SRMs for all measures and shows that the MHQ score [(ES = 0.79 95% CI: -1.64 to 3.32) and SRM = 0.56 (95% CI: -1.88 to 3.00)] was highly responsive to capturing improvements in self-reported hand and wrist function between baseline and 4 months. ESs and SRMs for EQ-5D (utility) and EQ-5D (VAS) were larger for the “improved” changes compared to the other categories. Overall, there were no consistent patterns at detecting changes to hand and wrist functioning between baseline and 4 months. The same analysis was repeated for changes between four and 12 months (Appendix 1). Results suggest more consistent patterns between all instruments with ESs and SRMs indicating less than moderate responsiveness to capturing improvement and worsening from 4 to 12 months. Estimates ranged from 0.35 (MHQ) to -0.34 (EQ-5D VAS).

The results for the analyses that used the perceived benefit/harm measure are summarised in Appendices 2 and 3 for the alternative follow-up periods and suggest that all instruments show small responsiveness (ES and SRM < 0.5) to perceived benefit/harm from the treatments between baseline and 4 months. The MHQ score as highly responsive to assessments of benefit or harm over the 12 month follow-up period (ES > 0.8].

## Discussion

This study compared the psychometric properties of generic HRQoL measures [EQ-5D (utility), EQ-5D (VAS), SF-12 (PCS), SF-12 (MCS), SF-6D (utility)] and a condition-specific (MHQ) PROM in a large sample of participants with RA of the hand. We examined the acceptability, construct validity, convergent validity, internal consistency, interpretability and responsiveness of these measures, as defined by the COSMIN checklist [26] and the checklist of assessment criteria published by Brazier and colleagues [27]. The reliability and validity of the MHQ has previously been established [13]. This study is the first to estimate the validity of the MHQ against an objective measures of pain troublesomeness. It further compared the MHQ with generic HRQoL instruments (EQ-5D, SF-12, SF-6D) to understand the strengths and weaknesses of each of these instruments in studies of RA of the hand.

High response rates to all measures included in the study, particularly for the EQ-5D, indicate the high acceptance of these instruments, by the individuals who completed and responded to the questionnaires, and their suitability for self-administration. The high response rates over the course of the SARAH study were achieved through completing measures face to face at a research clinic and also through follow-up mechanisms that included reminders sent to study participants by post or by telephone. The order in which measures were presented in the self-completion patient questionnaires (MHQ, EQ-5D, SF-12) might have influenced the response rates. Our findings from analyses of construct validity generally support the ability of all the measures used to discriminate between different levels of RA severity of the hands. Mean HRQoL or utility scores for all measures were significantly different between participants experiencing differing severity of RA pain. This finding is not in line with the study by Marra et al. [9], which found that of the Health Utilities Index 2 and 3 (HUI2, HUI3), EQ-5D, SF-6D, RA Quality of Life Questionnaire (RAQoL) and the Health Assessment Questionnaire (HAQ) only EQ-5D and SF-6D scores significantly differed by level of RA severity. Strong associations were observed in our study between the MHQ score and RA pain severity, followed in strength of association with RA pain severity by the EQ-5D (utility) and the SF-6D. In addition, our results suggest that the MHQ was highly responsive to assessments of benefit or harm over the 12 month follow-up period within the SARAH trial. Adams et al. [36] previously concluded that the EQ-5D is more responsive to deterioration in RA pain than the SF-6D and the SF-6D is more responsive to RA improvement than the EQ-5D. The physical component of the SF-12 had more consistent construct validity in our study than the mental health component of the measure, which is in agreement with the findings of Kosinskli et al. [37] in their validation study of the SF-36. Our findings with regards to the ability of the physical and mental health components of the SF-12 to discriminate between RA pain severity are also in agreement with the study by Linde et al. [38].

Our convergent validity analysis indicated that the MHQ score correlates most strongly with the EQ-5D (utility) score. The low level of correlation found between the SF-12 (MCS) and MHQ, and between the remaining PROMs, indicates that their respective constructs may be non-overlapping. The physical component of the SF-12 demonstrated the highest degree of inter-relatedness, especially with the EQ-5D. All measures under investigation displayed acceptable internal consistency as measured by Cronbach’s alpha values (>0.70).

The large ESs and SRMs for the MHQ indicates that this measure is very responsive at detecting **changes in self-reported hand and wrist functioning**; its responsiveness was followed by that for the EQ-5D (utility), SF-12 (PCS) and SF-6D. The SF-12 (MCS) could only moderately detect such changes. Overall, the measures, particularly the MHQ and EQ-5D, were more responsive at detecting improvement in external measures of health rather than worsening or no change. Our condition-specific instrument (MHQ) performed better at detecting patient-reported changes in external measures of health compared to the generic measures. This finding contradicts Linde and colleagues [38] who found no superiority in responsiveness of RA clinical measures (Rheumatoid Arthritis Quality of Life Scale (RAQoL) and Health Assessment Questionnaire (HAQ)) compared to the EQ-5D.

A possible weakness of this study is that due to data limitations, we were unable to assess the criterion validity, content validity and test-retest reliability of the measures. Also, some of the limitations of the analytical strategy are related to the use of total scores for many of the PROMs instead of using weighted methods (through, for example, factor analysis or Item Response Theory) [39].

Despite the study limitations, it should help to inform clinical researchers and health economists in this field in their selection of PROMs for use in their clinical and health economic evaluations. More specifically, the precision of trials in the context of RA where health outcomes are measured through a single instrument will be enhanced by evidence surrounding the psychometric properties of the alternative outcome measures evaluated in our study.

## Conclusions

In conclusion, the instruments evaluated in this study displayed varying psychometric properties in the context of RA of the hand. Our results extend beyond those of Harrison et al. [40], who previously proposed that at least one measure of HRQoL is included in studies of inflammatory arthritis. Our study revealed that of the study measures, the MHQ was most responsive at detecting change in indicators of RA pain severity, whilst the EQ-5D offered advantages over the SF-12 and its preference-based derivative (SF-6D) with respect to some psychometric properties. However, the selection of a preferred instrument in evaluative studies should ultimately depend on the relative importance placed on individual psychometric properties and the importance placed on generation of health utilities for economic evaluation purposes. Future studies are also needed to establish the generalizability of our findings for different hand conditions and different hand practices.

## Appendix 1

**Table 6:** Responsiveness of measures over time to self-reported hand and wrist functioning; 4 to 12 months

	n	T_4_	T_12_	Δ (95% CI)	SRM (95% CI)	ES (95% CI)
MHQ						
Improved	177	64.79	61.04	-3.75 (-6.19 to -1.31)	-0.13 (-2.57 to 2.31)	-0.19 (-2.63 to 2.25)
No change	150	56.97	58.53	1.56 (-1.11 to 4.23)	0.06 (-2.61 to 2.73)	0.09 (-2.58 to 2.76)
Worsened	113	45.37	46.22	0.85 (-2.12 to 3.82)	0.04 (-18.9 to 19.65)	0.05 (-2.92 to 3.02)
EQ-5D (utility)						
Improved	177	0.69	0.67	-0.02 (-0.05 to 0.01_	-0.06 (-0.09 to -0.03)	-0.09 (-0.12 to -0.06)
No change	150	0.62	0.64	0.02 (-0.02 to 0.06)	0.06 (0.03 to 0.10)	0.091 (0.05 to 0.13)
Worsened	113	0.48	0.57	0.09 (0.03 to 0.15)	0.22 (0.16 to 0.28)	0.31 (0.25 to 0.37)
EQ-5D (VAS)						
Improved	177	75.78	68.92	-6.86 (-9.25 to -4.47)	-0.24 (-2.63 to 2.14)	-0.35 (-2.74 to 2.03)
No change	150	67.46	68.55	1.09 (-1.95 to 4.13)	0.04 (-2.99 to 3.08)	0.06 (-2.98 to 3.10)
Worsened	113	60.43	58.66	-1.77 (-5.21 to 1.67)	-0.06 (-3.49 to 3.75)	-0.08 (-3.52 to 3.35)
SF-12 (PCS)						
Improved	177	36.63	34.19	-2.44 (-4.45 to -0.43)	-0.12 (-2.13 to 1.88)	-0.17 (-2.18 to 1.83)
No change	150	34.48	35.22	0.74 (-1.04 to 2.52)	0.05 (-1.74 to 1.83)	0.06 (-1.72 to 1.85)
Worsened	113	30.44	29.64	-0.8 (-2.84 to 1.24)	-0.05 (-2.09 to 1.99)	-0.07 (-2.11 to 1.97)
SF-12 (MCS)						
Improved	177	48.12	47.16	-0.96 (-3.22 to 1.31)	-0.04 (-2.31 to 2.22)	-0.17 (-2.17 to 1.84)
No change	150	49.29	50.81	1.52 (-0.50 to 3.54)	0.09 (-1.93 to 2.11)	0.13 (-1.89 to 2.15)
Worsened	113	44	44.96	0.96 (-1.69 to 3.61)	0.04 (-2.61 to 2.69)	0.06 (-2.59 to 2.72)
SF-6D						
Improved	177	0.68	0.64	-0.04 (-0.06 to -0.02)	-0.15 (-0.17 to -0.13)	-0.22 (-0.24 to -0.19)
No change	150	0.65	0.67	0.02 (0.00 to 0.04)	0.18 (0.09 to 0.13)	0.15 (0.13 to 0.18)
Worsened	113	0.59	0.6	0.01 (-0.02 to 0.04)	0.05 (0.02 to 0.07)	0.06 (0.04 to 0.09)

## Appendix 2

**Table 7:** Responsiveness of measures over time to perceived benefit/harm; baseline to 4 months

	n	T_0_	T_4_	Δ (95% CI)	SRM (95% CI)	ES (95% CI)
MHQ						
Benefit	303	51.91	57.86	5.95 (2.75 to 9.15)	0.21 (-1.70 to 3.12)	0.29 (-1.61 to 2.21)
No benefit	116	49.34	49.14	-0.2 (-3.46 to 3.06)	-0.01 (-2.98 to 2.96)	-0.02 (-2.98 to 2.95)
Harm	11	45.93	48.37	2.44 (-11.61 to 16.48)	0.10 (-7.40 to 7.61)	0.14 (-7.36 to 7.65)
EQ-5D (utility)						
Benefit	303	0.59	0.63	0.04 (0.00 to 0.08)	0.11 (0.08 to 0.14)	0.15 (0.13 to 0.18)
No benefit	116	0.59	0.57	-0.02 (-0.09 to 0.05)	-0.05 (-0.09 to 0.00)	-0.07 (-0.12 to -0.02)
Harm	11	0.55	0.59	0.04 (-0.23 to 0.31)	0.09 (-0.13 to 0.31)	0.13 (-0.09 to 0.35)
EQ-5D (VAS)						
Benefit	303	68.92	71.99	3.07 (0.32 to 5.82)	0.13 (-1.85 to 2.10)	0.18 (-1.80 to 2.15)
No benefit	116	65.77	63.09	-2.68 (-7.93 to 2.57)	-0.09 (-3.72 to 3.53)	-0.13 (-3.75 to 4.49)
Harm	11	61.18	61.81	0.63 (-18.12 to 19.38)	0.02 (-15.19 to 15.23)	0.03 (-15.18 to 15.23)
SF-12 (PCS)						
Benefit	303	34.02	35.27	1.25 (-0.64 to 3.14)	0.07 (-1.16 to 1.32)	0.11 (-1.14 to 1.35)
No benefit	116	32.85	33.59	0.74 (-1.95 to 3.43)	0.05 (-1.94 to 2.04)	0.07 (-1.92 to 2.06)
Harm	11	27.23	29.06	1.83 (-11.40 to 15.06)	0.08 (-9.47 to 9.63)	0.12 (-9.43 to 9.66)
SF-12 (MCS)						
Benefit	303	48.07	47.85	-0.22 (-2.39 to 1.96)	-0.01 (-1.46 to 1.44)	-0.02 (-1.47 to 1.43)
No benefit	116	47.45	47.93	0.48 (-2.89 to 3.85)	0.03 (-2.45 to 2.50)	0.04 (-2.43 to 2.51)
Harm	11	35.9	33.66	-2.24 (-19.75 to 15.27)	-0.08 (-12.62 to 12.46)	-0.11 (-12.64 to 12.43)
SF-6D						
Benefit	303	0.65	0.66	0.01 (-0.01 to 0.03)	0.05 (0.03 to 0.06)	0.06 (0.05 to 0.08)
No benefit	116	0.63	0.64	0.01 (-0.03 to 0.05)	0.05 (0.02 to 0.08)	0.07 (0.04 to 0.10)
Harm	11	0.61	0.52	-0.09 (-0.24 to 0.06)	-0.36 (-0.45 to -0.27)	-0.51 (-0.06 to -0.04)

## Appendix 3

**Table 8:** Responsiveness of measures over time to perceived benefit/harm; baseline to 12 months

	n	T_0_	T_12_	Δ (95% CI)	SRM (95% CI)	ES (95% CI)
MHQ						
Benefit	276	51.15	57.83	6.68 (3.75 to 9.61)	0.26 (-1.74 to 2.27)	1.03 (-0.97 to 3.04)
No benefit	110	50.86	49.15	-1.71 (-6.21 to 2.79)	-0.07 (-3.18 to 3.05)	1.02 (-2.09 to 4.14
Harm	6	46.33	46.34	0.01 (-20.65 to 20.67)	0.00 (-11.13 to 11.13)	1.22 (-9.91 to 12.35)
EQ-5D (utility)						
Benefit	276	0.59	0.61	0.06 (0.02 to 0.10)	0.17 (0.14 to 0.19)	0.24 (0.20to 0.26)
No benefit	110	0.59	0.57	-0.03 (-0.09 to 0.03)	-0.09 (-0.14 to -0.05)	-0.13 (-0.17 to -0.08)
Harm	6	0.53	0.54	-0.05 (-0.03 to 0.23)	-0.15 (-0.25 to -0.03)	-0.22 (-0.34 to -0.09)
EQ-5D (VAS)						
Benefit	276	69.57	67.92	-1.65 (-3.67 to 0.37)	-0.06 (-2.08 to 1.96)	-0.08 (-2.10 to 1.94)
No benefit	110	64.89	61.3	-3.59 (- 7.08 to -0.09)	-0.13 (-3.63 to 3.35)	-0.19 (-3.68 to 3.30)
Harm	6	60.16	57	-3.16 (-19.83 to 13.56)	-0.11 (-16.83 to 16.61)	-0.15 (-16.87 to 18.42)
SF-12 (PCS)						
Benefit	276	34.44	32.93	-1.51 (-2.77 to -0.24)	-0.08 (-1.35 to 1.18)	-0.12 (-1.38 to 1.15)
No benefit	110	31.79	31	-0.79 (-2.86 to 1.28)	-0.15 (-2.13 to 2.02)	-0.08 (-2.15 to 1.99)
Harm	6	24.84	36.08	11.24 (-0.27 to 22.75)	0.67 (-10.84 to 12.19)	0.99 (-10.52 to 12.51)
SF-12 (MCS)						
Benefit	276	48.26	47.31	-0.95 (-2.44 to 0.54)	-0.05 (-1.54 to 1.45)	-0.06 (-1.56 to 1.42)
No benefit	110	48.04	49.44	1.4 (-1.06 to 3.86)	0.08 (-2.38 to 2.54)	0.12 (-2.34 to 2.57)
Harm	6	32.7	37.74	5.04 (-11.59 to 21.67)	0.21 (-16.42 to 16.84)	0.31 (-16.32 to 16.95)
SF-6D						
Benefit	276	0.64	0.63	-0.01 (-0.03 to 0.01)	-0.04 (-0.06 to -0.02)	-0.06 (-0.07 to -0.04)
No benefit	110	0.63	0.63	0 (-0.02 to 0.02)	0 (-0.02 to 0.02)	0 (-0.02 to 0.02)
Harm	6	0.58	0.58	0 (-0.06 to 0.06)	0 (-0.06 to 0.06)	0 (-0.06 to 0.06)

## Abbreviations

ADL: Activities of daily living
CI: CONFIDENCE interval
COSMIN: Consensus-based Standards for the selection of health Measurement Instruments
EQ-5D: EuroQol EQ-5D
ES: Effect Size
HAQ: Health Assessment Questionnaire
HRQoL: Health-related quality of life
HUI: Health Utility Index
MCS: Mental Health Component Score
MHQ: Michigan Hand Outcome Questionnaire
NRAS: National Rheumatoid Arthritis Society
PCS: Physical Component Summary Score
PROMS: Patient Reported Outcome Measures
QALYs: Quality-adjusted life years
RA: Rheumatoid arthritis
RAQoL: Rheumatoid Arthritis Quality of Life Scale
SD: Standard deviation
SF-12: Short Form 12 Dimensions
SF-6D: Short Form 6 Dimensions
SRM: Standardised response means
VAS: Visual Analogue Scale
